# Laryngopharyngeal Reflux Disease: Outcome of Patients After Treatment in Otolaryngology Clinics

**DOI:** 10.7759/cureus.12195

**Published:** 2020-12-21

**Authors:** Montasir Junaid, Sadaf Qadeer Ahmed, Maliha Kazi, Hareem U Khan, Muhammad Sohail Halim

**Affiliations:** 1 Otolaryngology - Head and Neck Surgery, Armed Forces Hospital Southern Region, Khamis Mushait, SAU; 2 Otolaryngology - Head and Neck Surgery, Sir Syed College of Medical Sciences, Karachi, PAK; 3 Otorhinolaryngology, Manchester Royal Infirmary, Karachi, PAK; 4 Otolaryngology - Head and Neck Surgery, Stanford University, Palo Alto, USA; 5 Byers Eye Institute, Stanford University, Palo Alto, USA

**Keywords:** rfs, rsi, laryngopharyngeal reflux disease

## Abstract

Introduction

Laryngopharyngeal reflux (LPR) is a different entity from gastroesophageal reflux disease (GERD). Patients with LPR usually present with a variety of symptoms such as hoarseness, voice fatigue, burning sensation in the throat, persistent cough, sore throat, dysphagia, a sensation of a lump in the throat, and chronic throat clearing. The management of LPR is based on medications (proton pump inhibitors) along with lifestyle and dietary modifications. It has been suggested that the Reflux Symptom Index (RSI) and Reflux Finding Score (RFS) are useful parameters to assess patients with LPR.

The aim of this study is to assess the subjective and objective benefits of RFS and RSI for diagnosing and management of LPR in the tertiary care center and to find the difference in RSI and RSI scoring with respect to gender.

Methods

A prospective study was performed and 102 patients were included according to inclusion criteria. RFS and RSI questionnaires were filled on the first visit of patients and then treatment with proton pump inhibitors was started along with lifestyle modification instructions. Questionnaires were filled after four weeks and then 12weeks post-treatment. Repeated measure analysis of variance (ANOVA) was performed to compare the mean RFS and RSI from baseline to the end of treatment. The post hoc analysis was done using the Bonferroni test of multiple comparisons. An independent sample t-test was also used to compare the mean RFS and RSI between genders. P-values less than 0.05 were considered statistically significant

Results

RFS and RSI were found to be significantly decreased post-treatment after four weeks and 12 weeks post-treatment (p-value- <0.01). Eight point eight percent (8.8%) side effects were observed in the study, the change in quality of life after a three-month treatment was significantly improved among 62.7% patients, and 75.5% did lifestyle modifications. In the mean comparison of RFS and RSI with respect to gender, it was observed that the mean RFS of females samples after one month and three months of treatment were significantly less as compared to male samples, p<0.01. There was no significant mean difference observed for RSI after one month and three months of treatment with respect to gender (p>0.05).

Conclusion

RFS and RSI are convenient and helpful for diagnosing LPR, and they can be easily implemented in ear, nose, throat (ENT) clinics for the subjective and objective assessment of LPR. Females showed greater improvement on laryngoscopy findings (RFS scores) post-treatment as compared to males.

## Introduction

Laryngopharyngeal reflux disease (LPR), a recently described terminology [1], occurs as a result of the retrograde flow of gastric contents into the laryngopharynx. It is a different entity from gastroesophageal reflux disease (GERD), the latter is caused by the backflow of gastric contents into the esophagus, which leads to tissue damage or esophagitis and heartburn. The most significant difference is that the majority of patients with LPR do not have esophagitis or heartburn [2]. In numerous studies, the incidence of heartburn in patients with LPR is less than 40% and the incidence of esophagitis is approximately 25% [1]. Patients with LPR usually present with a variety of symptoms such as hoarseness (which is the most common complaint, voice fatigue, burning sensation in the throat, persistent cough, sore throat, dysphagia, the sensation of a lump in the throat, and chronic throat clearing [3].

The diagnosis of LPR is based on clinical symptoms and physical examination, with or without pH manometry. Nevertheless, pH manometry is still considered by many as a more accurate assessment of LPR. However, there is inconsistency in the methodology of pH monitoring in much of the published literature, in particular, the location of the upper probe [4-5], the impact of diet [6], the role of nonacidic factors in LPR [7-8], and, importantly, the correlation of pH findings with signs and symptoms. Whether this test will ever become widely used in routine clinical care remains doubtful, given the invasive nature of the time-consuming procedures, their limited availability, and the expertise required. Therefore, clinical markers for the diagnosis and management of this disease are needed.

The management of LPR is based on medications (proton pump inhibitors) along with lifestyle and dietary modifications. It has been suggested that the Reflux Symptom Index (RSI) and Reflux Finding Score (RFS) may be useful parameters [9-10]. The RSI has been designed to raise the clinical suspicion of LPR in patients presenting with ears, nose, and throat (ENT) symptoms, whereas the RFS has been designed to characterize the morphologic lesions presumably associated with LPR [11]. It has remained unclear, however, as of today whether the results of RSI and RSF can be standardized to guide the treatment of suspected LPR.

In this study, we will use RFS and RSI for the diagnosis and management of laryngopharyngeal reflux disease. We will evaluate the symptoms and signs resolution after one month and then three months of acid-suppressive therapy with 40 mg esomeprazole in ENT patients who will be selected for the treatment based on abnormal results of RSI and RFS.

In our tertiary care center, the RSI and RFS were not regularly used. The aim of the study was to assess the subjective and objective benefits of RFS and RSI for diagnosing and management of LPR. This study does not only benefit in the proper treatment of patients but also helps in the comparison before and after medical therapy for the disease.

## Materials and methods

After ethical review committee approval, a prospective study was initiated on patients who came to ENT clinics and were diagnosed as laryngopharyngeal reflux (LPR) based on the Reflux Symptom Index (RFS) and Reflux Finding Score (RFS) questionnaires. After informed consent, the diagnosed patients were included in the study with age greater than 18 years while patients who had prior surgery for gastroesophageal reflux disease or received any medical treatment previously, were unwilling to participate in the study, had a history of neurological illness, co-morbidities such as asthma, chronic obstructive pulmonary disease (COPD), or any other laryngeal pathologies were excluded from the study. A total of 102 patients were included over the period of one year (2019-2020) in the study after sample size calculation.

Management

After the diagnosis of LPR, treatment was initiated with proton pump inhibitors (omeprazole 40 mg once a day for 12 weeks). Along with that, patients were given written instructions for lifestyle modifications, which included taking dinner four hours prior to bedtime, drinking at least 12 glasses of water daily, usage of yogurt or milk with each meal, avoidance of oily, spicy, and fried items in meals, cessation of smoking, and 30 minutes daily physical activity.

Scoring

The RSI and RFS questionnaires 9, 10 were used for scoring. Patients were asked to fill the RSI questionnaire at the first visit and if scores came greater than or equal to 13 then they were included in the study and the RFS Questionnaire was filled by the primary physician after performing fiberoptic laryngoscopy, and if scores came greater than or equal to 7 then patients were considered as positive for LPR disease and became part of the study (Tables 1-2).

**Table 1:** Reflux Finding Score (RFS)

Pseudosulcus	0 = absent; 2 = present
Ventricular obliteration	0 = no; 2 = partial; 4 = total
Erythema/hyperemia	0 = no; 2 = arytenoids; 4 = diffuse
Edema of the vocal cords	0 = no; 1 = medium; 2 = moderate; 3 = severe; 4 = polypoid
Diffuse laryngeal edema	0 = no; 1 = medium; 2 = moderate; 3 = severe; 4 = obstructive
Hypertrophy of posterior commissure	0 = no; 1 = medium; 2 = moderate; 3 = severe; 4 = obstructive
Granuloma/granulation	0 = absent; 2 = present
Dense endolaryngeal mucous	0 = absent; 2 = present

**Table 2:** Reflux Symptom Index (RSI)

Hoarseness or a problem with your voice	0	1	2	3	4	5
Clearing your throat	0	1	2	3	4	5
Excess throat mucus or feeling of postnasal drip	0	1	2	3	4	5
Difficulty swallowing food, liquids, or tablets	0	1	2	3	4	5
Coughing after eating or lying down	0	1	2	3	4	5
Breathing difficulties or choking episodes	0	1	2	3	4	5
Troublesome or annoying cough	0	1	2	3	4	5
Sensation of something sticking in your throat or of a lump in your throat	0	1	2	3	4	5
Heartburn, indigestion, or stomach acid coming up (dyspepsia component mentioned in the text)	0	1	2	3	4	5

The scores were noted at the first visit to the ENT clinic, the treatment was initiated, patients were then followed up after one month and then the third month post-treatment for evaluation while questionnaires were filled at both visits again for comparison.

Statistical analysis

Data were stored and analyzed using the Statistical Package for the Social Sciences (SPSS) version 23.0 (IBM Corp., Armonk, NY). Mean with standard deviation was given for quantitative data sets like age, RFS, and RSI. Counts with percentages were reported for qualitative data sets like gender, smoking, presenting complaint, observed side effects, and change in the quality of life after three months of treatment and lifestyle modification. Repeated measure analysis of variance (ANOVA) was performed to compare the mean RFS and RSI from baseline to the end of treatment. The post hoc analysis was done using the Bonferroni test of multiple comparisons. An independent sample t-test was also used to compare the mean RFS and RSI between genders. P-values less than 0.05 were considered statistically significant. Pie diagrams, line charts, and bar diagrams were used to graphically present data.

## Results

**Figure 1:**
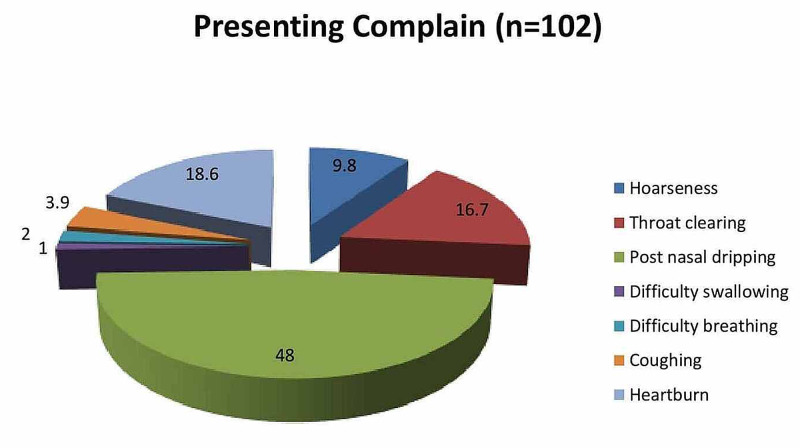
Pie chart of presenting complaints

Table 3 reports the baseline characteristics of the studied samples. In the present study, there were 102 patients having a mean age of 41.8 (SD=±10.1) years. The majority (56.9%) were of the female gender. There were 31.4% of patients found with tobacco smoking. Out of the total, 48% had complaints of post-nasal dripping, 18.6% had complaints of heartburn, and 16.7% had complaints of throat-clearing (Figure 1). 

**Table 3:** Baseline characteristics of studied samples (n=102)

Characteristics		n	%
Age	Mean ± SD	41.8	±10.1
Gender	Male	44	43.1
	Female	58	56.9
Smoking	Tobacco smoking	32	31.4
	No smoking	70	68.6
Presenting Complaint	Hoarseness	10	9.8
	Throat clearing	17	16.7
	Post nasal dripping	49	48.0
	Difficulty swallowing	1	1.0
	Difficulty breathing	2	2.0
	Coughing	4	3.9
	Heartburn	19	18.6

Table 4 reports the mean and standard deviation for RFS and RSI at three levels of study. The results showed that the mean RFS before treatment was 9.53 (SD=±1.87); after one month of treatment, it was 2.89 (SD=±1.02); and after three months of treatment, it went down to 2.79 (SD=1.07). The decrease in mean RFS from baseline to the end of treatment was considered statistically significant, with p<0.01. Whereas the mean RSI before treatment was 18.17 (SD=±4.06), after one month of treatment, it was 6.67 (SD=±2.11), and after three months of treatment, it went down to 6.27 (SD=1.97). The decrease in mean RSI from baseline to the end of treatment was also considered statistically significant, with p<0.01.

**Table 4:** Mean comparison of RFS and RSI using repeated measure ANOVA RFS: Reflux Finding Score; RSI: Reflux Symptom Index; ANOVA: analysis of variance

Parameters	Before Treatment		After One Month		After Three Months		p-value
	Mean	SD	Mean	SD	Mean	SD	
RFS	9.53	1.87	2.89	1.02	2.79	1.07	<0.01*
RSI	18.17	4.06	6.67	2.11	6.27	1.97	<0.01*
*p<0.05 was considered statistically significant using repeated measure ANOVA							

Table 5 gives the post hoc analysis for RFS and RSI using the Bonferroni test of multiple comparisons. The results showed that the decrease in RFS after one month from baseline was 6.67 units, which is considered statistically significant with p<0.01, the decrease in RFS after three months from baseline was 6.67 units, which was found statistically significant, with p<0.01; however, the mean difference of RFS from one month to three months of treatment was 0.09 units, which was found statistically insignificant (p=0.34). Similarly, the mean difference of RSI from baseline to after one month of treatment was 11.5 units and after three months, it was 11.9 units, whereas the mean difference of RSI from one month to three months of treatment was 0.40 units but all were found statistically significant, with p<0.01.

**Table 5:** Post hoc analysis of RFS and RSI using the Bonferroni test of multiple comparisons RFS: Reflux Finding Score; RSI: Reflux Symptom Index

Parameters	Comparison of	Comparison With	Mean Difference	SE	p-value
RFS	Before treatment	After one month	6.64	0.14	<0.01*
		After three months	6.74	0.16	<0.01*
	After one month	After three months	0.09	0.06	0.34
RSI	Before treatment	After one month	11.5	0.33	<0.01*
		After three months	11.9	0.40	<0.01*
	After one month	After three months	0.40	0.10	<0.01*

Table 6 reports there were 8.8% side effects observed in the study, change in the quality of life after three-month treatment was significantly improved among 62.7% samples, and 75.5% modified their lifestyle.

**Table 6:** Outcomes on side effects*, change in quality of life, and lifestyle modification

		n	%
Observed side effects*	Yes	9	8.8
	No	93	91.2
Change in quality of life after 3 months of treatment	Significantly improved	64	62.7
	Some improvement	38	37.3
	No improvement	0	0
Lifestyle modification	Yes	77	75.5
	Followed instruction irregularly	25	24.5

**Figure 2:**
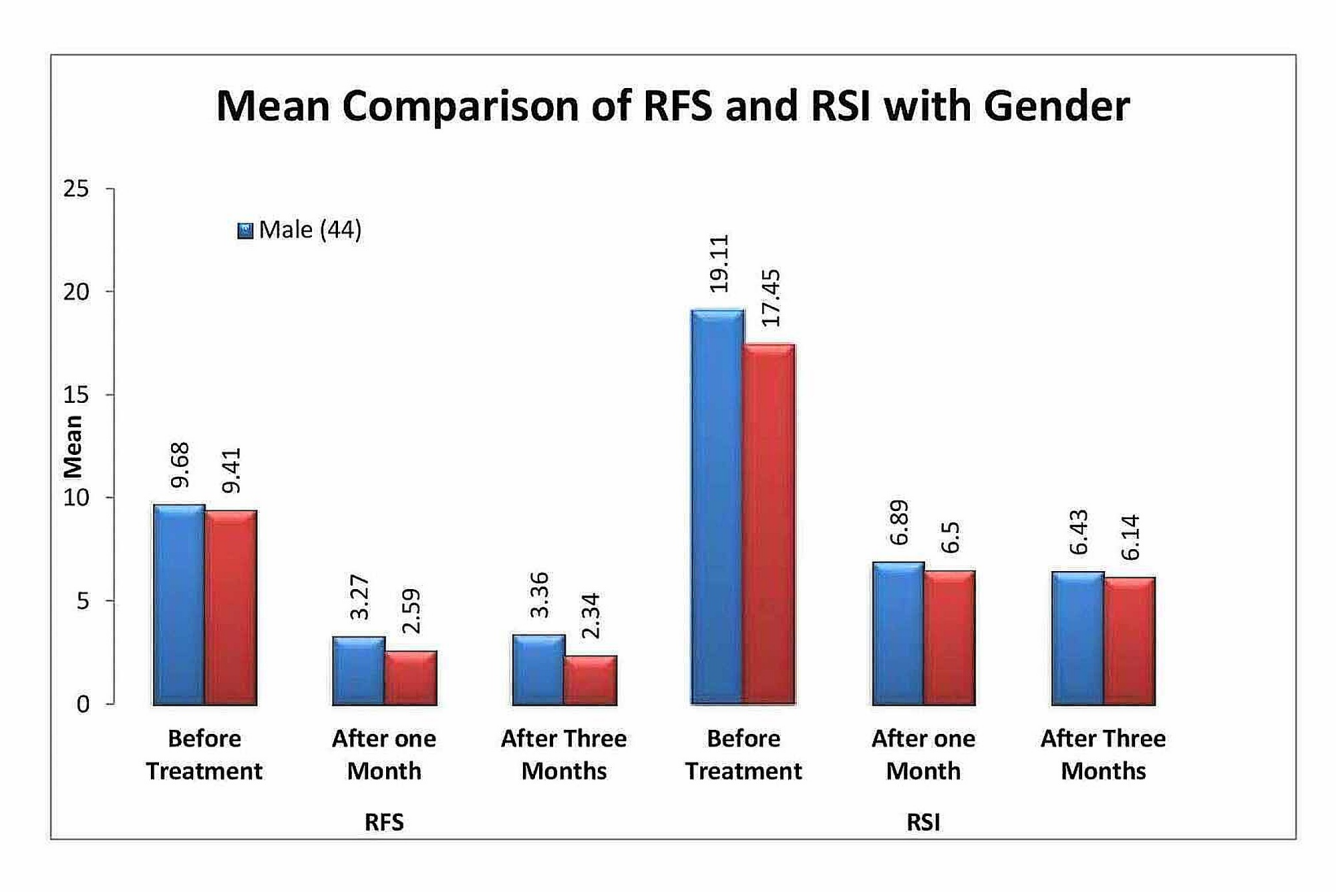
Bar chart showing mean comparison of RFS and RSI with respect to gender RFS: Reflux Finding Score; RSI: Reflux Symptom Index

Table 7 gives the mean comparison of RFS and RSI with respect to gender, it was observed that the mean RFS of female samples after one month and three months of treatment were significantly less as compared to male samples (p<0.01) and there was no significant mean difference observed for RSI after one month and three months of treatment with respect to gender (p>0.05) (Figure 2).

**Table 7:** Mean comparison of RFS and RSI with respect to gender RFS: Reflux Finding Score; RSI: Reflux Symptom Index

Parameters	Gender	Mean	SD	p-value
RFS score before treatment	Male (44)	9.68	1.84	0.47
	Female (58)	9.41	1.89	
RFS after 1 month treatment	Male (44)	3.27	1.18	<0.01*
	Female (58)	2.59	0.75	
RFS after 3 months treatment	Male (44)	3.36	1.05	<0.01*
	Female (58)	2.34	0.84	
RSI score before treatment	Male (44)	19.11	4.2	0.04*
	Female (58)	17.45	3.8	
RSI after 1 month treatment	Male (44)	6.89	2.30	0.36
	Female (58)	6.50	1.93	
RSI after 3 months treatment	Male (44)	6.43	2.42	0.45
	Female (58)	6.14	1.55	
*p<0.05 was considered significant using the independent sample t-test				

**Figure 3:**
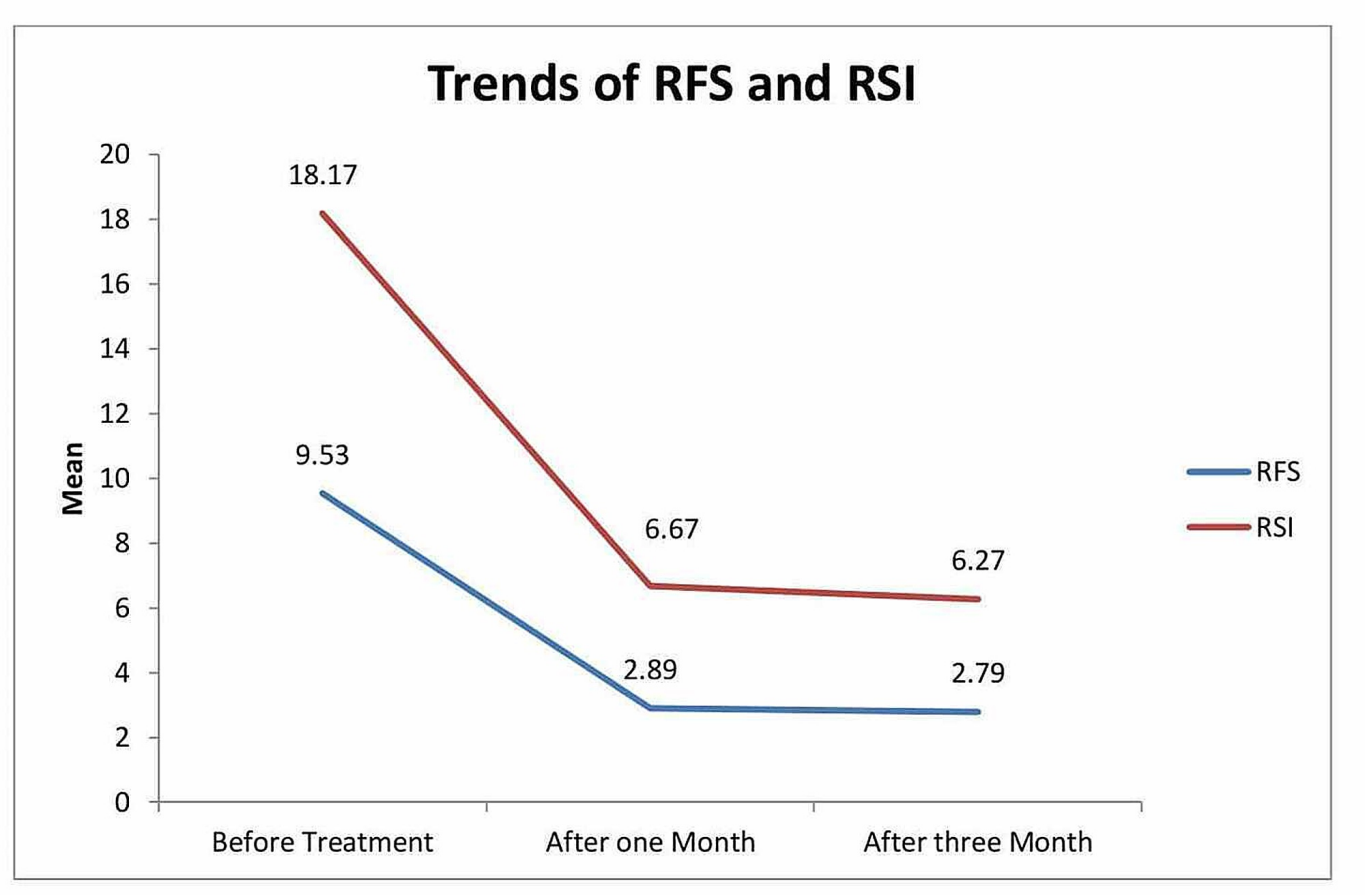
Trends of RFS and RSI RFS: Reflux Finding Score; RSI: Reflux Symptom Index

Figure 3 shows a line chart showing a significant decrease in RFS and RSI from baseline to the third month of treatment in studied samples.

## Discussion

With recent advancements, the diagnosis of reflux has become quite challenging. It is a distinguished entity from gastroesophageal reflux disease (GERD) and should be managed by otolaryngologists.

In our study, we found the mean age of patients who were diagnosed with LPR was 41.8 (SD=±10.1) years, which was comparable with studies in the published literature [12]. While most of the patients were female, i.e. 56.9%, which is similar to the study conducted by Pokharel et al. [13] and Haruma et al. [14].

In our study group, the most common complaint was excess throat mucus or postnasal dripping (48%) in contrast to other studies where globus sensations, hoarseness, and coughing were the main concerns for visits to otolaryngologists [15-17].

The patients who were identified and diagnosed as suspected LPR were given the RSI questionnaire to be filled by them and their laryngoscope findings were filled by the attending physician on their first visit. The patients were then started on medical management with proton pump inhibitors (40 mg daily) and lifestyle modifications were explained fully along with written instructions. The patients were asked to visit the ENT clinic after four weeks and 12 weeks. The questionnaires were again filled at the 4th and 12th weeks. The RFS and RSI showed significant improvement after initiation of treatment at the 4th week and 12th week, respectively, and the p-value was found to be significant (< 0.01). The study results were found comparable with Belafsky et al. [9-10] and Silva et al. [12]. Belafsky continued treatment for a period of six months, whereas, in our study, treatment was given for three months.

Though in literature, RFS and RSI have been questioned for not including various ENT-associated signs and symptoms [18-20], on the contrary, there are several studies supporting the use of RSI and RFS as reliable indicators for the diagnosis and treatment of LPR [21-22].

In order to keep the patients' perspective in mind, along with the RFS and RSI questionnaire to be filled in the proforma, the subjective assessment was measured after three months by asking about the side-effects of the medications and any change in the quality of life post-treatment. Patients were also asked specifically if lifestyle modifications helped in their improvement.

Out of 102 patients, eight patients complained of side effects, which included a mild nauseous feeling that settled later in the day; one patient complained of abdominal bloating for three days after the initiation of treatment, which improved afterward.

Nearly all patients reported an improvement in the quality of life, 64 patients reported significant improvement, and none of the patients reported a decline or no improvement post-treatment. Similar to the study by Siupsinskiene et al. [23], which showed a significant improvement in the quality of life after proton pump inhibitors therapy in LPR while in another study by Habermann et al. [22], 41% of patients reported improvement in the quality of life followed by significant improvement 31% while 13% patients reported no change or worse quality of life, which was contrary to our study where no patient reported worse or poor quality of life post-treatment.

Lifestyle modifications were advised concomitantly with the medications to the patients as it has a positive and greater impact on management overall [16]. Out of the total, 75 patients followed instructions carefully but 25 patients were not fully compliant with the written instructions. Though patients who followed instructions along with medications reported significantly improved quality of life post-treatment, it was not the objective of this study hence not estimated.

In the literature, gender has remained the topic of debate in laryngopharyngeal reflux disease. Few studies showed female predominance [13-14,24] in LPR while others reported males having LPR more commonly [25]. It is still unclear whether there are differences in the severity of symptoms or objective signs on laryngoscope findings among gender. In our study, the mean of RFS and RSI was compared with respect to gender and it was found out that RFS was significantly lowered after the 1st and 3rd month post-treatment in females in comparison with males. This could be due to mucosal changes among gender, which is yet to be proven. RSI showed no significant difference between the two groups.

The limitation can be the small sample size and, in this study, no comparison with ambulatory 24-hour double probe monitoring has been done due to the invasive nature and cost of the test.

The strength of the study is that RFS is filled and flexible endoscopy has been performed by a single attending physician in order to reduce inter-observer bias. This study is the first of its kind where RFs and RSI scores are compared between male and female gender in order to find any significant difference. Further study with a large sample size is recommended to validate in this regard.

## Conclusions

RFS and RSI are convenient and helpful in diagnosing LPR, and they can be easily implemented in ENT clinics for the subjective and objective assessment of LPR. Its use may prevent the unnecessary costs of invasive laboratory studies and imaging. Females showed greater improvement on laryngoscopy findings (RFS scores) post-treatment as compared to males.
